# Green Tea Catechin Plus Inulin Improves Insulin Resistance Without Reducing Visceral Fat and Shows Exploratory Gut Microbiota Signals in Adults with Visceral Obesity: A Double-Blind Randomized Controlled Trial

**DOI:** 10.3390/nu18050851

**Published:** 2026-03-06

**Authors:** Chikara Iino, Keita Mikami, Keisuke Furusawa, Satoshi Sato, Kenta Yoshida, Tohru Yamaguchi, Hiroto Bushita, Keita Kinoshita, Yuji Matsui, Seiya Imoto, Takuro Iwane, Yoshinori Tamada, Koichi Murashita, Shigeyuki Nakaji, Tatsuya Mikami, Hirotake Sakuraba

**Affiliations:** 1 Department of Gastroenterology, Hematology, and Clinical Immunology, Hirosaki University Graduate School of Medicine, Hirosaki 036-8562, Japan; km_1522@hirosaki-u.ac.jp (K.M.); fk-9562@hirosaki-u.ac.jp (K.F.); satoshis@hirosaki-u.ac.jp (S.S.); kyoshida@hirosaki-u.ac.jp (K.Y.); hirotake@hirosaki-u.ac.jp (H.S.); 2 Human Health Care Products Research Laboratories, Kao Corporation, Tokyo 131-8501, Japan; yamaguchi.tohru@kao.com (T.Y.); bushita.hiroto@kao.com (H.B.); kinoshita.keita@kao.com (K.K.); matsui.yuji@kao.com (Y.M.); 3 Research Center for Health-Medical Data Science, Hirosaki University Graduate School of Medicine, Hirosaki 036-8562, Japan; y.tamada@hirosaki-u.ac.jp; 4 Department of Active Life Promotion Sciences, Hirosaki University Graduate School of Medicine, Hirosaki 036-8562, Japan; 5 Human Genome Center, Institute of Medical Science, University of Tokyo, Tokyo 108-0071, Japan; imoto@ims.u-tokyo.ac.jp; 6 Research Institute of Health Innovation, Hirosaki University, Hirosaki 036-8562, Japan; i-tak@hirosaki-u.ac.jp (T.I.); murasita@hirosaki-u.ac.jp (K.M.); 7 Department of Preemptive Medicine, Innovation Center for Health Promotion, Hirosaki University Graduate School of Medicine, Hirosaki 036-8562, Japan; nakaji@hirosaki-u.ac.jp (S.N.); tmika@hirosaki-u.ac.jp (T.M.)

**Keywords:** green tea catechin, inulin, visceral fat, insulin resistance, HOMA-IR, gut microbiota, *Coprococcus*, butyrate, obesity

## Abstract

**Background:** Green tea catechins and inulin may improve metabolic health and modulate the gut microbiota; however, double-blind trials evaluating visceral fat, insulin resistance, and microbiota concurrently are limited. **Methods:** We conducted a double-blind, parallel-group, randomized, placebo-controlled trial in Japanese adults aged 20–75 years with visceral fat area (VFA) ≥ 80 cm^2^ and BMI ≥ 23 kg/m^2^ (trial registration: Japan Registry of Clinical Trials (jRCT), jRCTs021230004 (registered 16 May 2023)). Participants were randomized to a catechin + inulin beverage (catechins 400 mg/day; inulin 2.3 g/day) or placebo for 12 weeks. The primary outcome was the change in VFA (bioelectrical impedance). Secondary outcomes included HOMA-IR, metabolic markers, liver fat assessed by the controlled attenuation parameter (CAP), and genus-level gut microbiota. **Results:** Ninety-six participants were randomized (catechin + inulin, n = 49; placebo, n = 47); 47 and 44, respectively, were included in the full analysis set for the primary outcome. The 12-week change in VFA did not differ between groups (mean change: +0.91 vs. +4.61 cm^2^; *p* = 0.243). HOMA-IR decreased in the catechin + inulin group and increased in the placebo group, yielding a significant between-group difference (mean change: −0.32 vs. +0.18; *p* = 0.020). No other secondary outcomes showed significant between-group differences. In exploratory microbiota analyses, no genus remained significant after false discovery rate correction; however, *Coprococcus* and *Bifidobacterium* showed nominal between-group differences (unadjusted *p* < 0.05). Changes in *Coprococcus* were inversely correlated with changes in HOMA-IR (r = −0.28; *p* = 0.010). **Conclusions:** Catechin plus inulin for 12 weeks did not reduce visceral fat but was associated with improved insulin resistance. Exploratory analyses suggest a potential association between increased *Coprococcus* and improved HOMA-IR, warranting further investigation.

## 1. Introduction

Visceral fat accumulation is strongly implicated in the development and progression of insulin resistance, type 2 diabetes, hypertension, dyslipidemia, and other obesity-related complications. In addition, visceral adipose tissue is increasingly recognized as an independent risk factor for cardiovascular events and all-cause mortality, beyond overall adiposity [1,2]. Therefore, dietary and lifestyle interventions targeting visceral fat are of particular clinical interest in the management of obesity and obesity-related metabolic disorders.

Green tea catechins are polyphenols abundantly found in green tea and have been reported to exhibit anti-obesity and metabolic effects through multiple mechanisms, including antioxidant activity, stimulation of fatty acid oxidation, inhibition of fat absorption, and an increase in energy expenditure. Randomized controlled trials (RCTs) using catechin-rich green tea extracts or catechin-fortified beverages have demonstrated that approximately 12 weeks of daily intake can reduce body weight and body fat, particularly abdominal visceral fat area (VFA), and concomitantly lower systolic blood pressure and LDL cholesterol [3,4,5]. Furthermore, clinical trials and meta-analyses in individuals with impaired glucose tolerance or type 2 diabetes suggest that green tea or catechin intake may modestly improve fasting glucose, glycated hemoglobin (HbA1c), and HOMA-IR levels [6,7].

Inulin is a water-soluble dietary fiber naturally present in plants such as chicory root and functions as a prebiotic because it resists digestion in the small intestine and is fermented by gut microbiota in the colon. Supplementation with inulin or inulin-type fructans (ITFs) has been reported to selectively increase beneficial bacteria such as *Bifidobacterium* spp., enhance the production of short-chain fatty acids (SCFAs), and thereby reduce body weight, BMI, body fat mass, and waist circumference, as well as improve fasting glucose, HbA1c, and HOMA-IR levels in several RCTs and meta-analyses [8,9]. Many of these trials have used daily doses of inulin in the range of approximately 10 g or more for at least 8–12 weeks, and dose-dependent effects on obesity-related outcomes have been suggested [8,9].

Growing evidence indicates that gut microbiota and their metabolites, especially SCFAs, play an important role in the pathophysiology of obesity and related metabolic diseases. Butyrate serves as a major energy source for colonocytes and, via G-protein–coupled receptors such as GPR41 and GPR43, promotes glucagon-like peptide-1 (GLP-1) secretion, enhances hepatic fatty acid oxidation, suppresses gluconeogenesis, and improves insulin sensitivity [10]. Reduced SCFA concentrations and depletion of butyrate-producing bacteria have been observed in obesity and type 2 diabetes [10,11], whereas dietary interventions with fermentable fibers that increase SCFA production are often associated with improvements in insulin resistance and inflammatory markers [8,9,10]. On this basis, combining green tea catechins, which promote fat oxidation and inhibit fat absorption, with inulin, which modulates gut microbiota and SCFA production, may exert additive or synergistic effects on visceral adiposity and insulin resistance.

A double-blind RCT from Taiwan tested a drink containing catechin-rich green tea with added inulin in overweight adults and reported significant reductions in body weight and body fat after 6 weeks of intake, with partial maintenance of the effect even 2 weeks after cessation [12]. This trial suggests that the combination of catechins and inulin may be useful for weight management. In Japanese adults, a trial combining a specific probiotic (*Bifidobacterium animalis* ssp. *lactis* GCL2505) with dietary fiber showed a significant reduction in visceral fat area measured by computed tomography (CT) [13]. However, human intervention studies that combine catechins and inulin remain scarce, and very few have evaluated VFA as the primary endpoint while simultaneously assessing insulin resistance, liver fat, and gut microbiota. In addition, whereas many inulin trials have used ≥10 g/day, evidence on more realistic, lower daily doses of inulin in combination with catechins is limited [8,9]. The combination of green tea catechins and inulin was selected based on previous evidence suggesting their potential benefits for metabolic health in overweight adults [12]. Mechanistically, green tea catechins have been shown to enhance fat oxidation and energy expenditure, while inulin acts as a prebiotic that modulates gut microbiota and promotes the production of short-chain fatty acids (SCFAs). We hypothesized that the co-administration of these two components might exert additive or synergistic effects on reducing visceral fat and improving metabolic markers through these distinct but complementary pathways.

Therefore, we aimed to evaluate the effects of 12-week daily intake of a beverage containing green tea catechins (400 mg/day) plus inulin (2.3 g/day) on VFA, insulin resistance, and gut microbiota in Japanese adults with visceral obesity. The catechin dose was selected to be within the range used in prior human studies evaluating visceral fat and metabolic outcomes, while the inulin dose reflects a practical, beverage-compatible amount intended for daily long-term intake in this double-blind, randomized, placebo-controlled trial; the primary endpoint was the change in VFA from baseline to week 12. Secondary endpoints included changes in insulin resistance (HOMA-IR), liver fat assessed by CAP, and gut microbiota composition.

## 2. Materials and Methods

### 2.1. Study Design and Participants

This study was a double-blind, randomized, placebo-controlled, parallel-group trial entitled “Intervention Study for Reduction of Visceral Fat by Tea Catechins and Inulin”. This trial was registered in the Japan Registry of Clinical Trials (jRCT; trial ID: jRCTs021230004) on 16 May 2023 (https://jrct.mhlw.go.jp/en-latest-detail/jRCTs021230004, accessed on 2 March 2026). Japanese men and women aged 20–75 years were recruited from the community. Key inclusion criteria were VFA ≥ 80 cm^2^, measured by bioelectrical impedance analysis, and BMI ≥ 23 kg/m^2^ at baseline. Major exclusion criteria included a change in body weight of ±2 kg or more during the 3 months prior to enrollment, serious hepatic, renal, or cardiovascular disease, use of medications or supplements that could strongly affect body weight or glucose metabolism, and any other condition judged by the investigators to interfere with participation.

Eligible participants were randomly assigned in a 1:1 ratio to receive either a test beverage containing green tea catechins and inulin (catechin + inulin group) or a placebo beverage (placebo group). Randomization was conducted by an independent third party using a stratified allocation procedure with sex, age, baseline VFA, and HbA1c as stratification factors. Strata were defined by sex (male/female) and by dichotomizing age, baseline VFA, and HbA1c at their respective medians. Within each stratum, allocation was performed using a permuted block design (block size = 2). Randomization sequences were generated by a computer program and implemented by an independent person responsible for allocation. Group assignments were concealed from participants, investigators, outcome assessors, and data analysts throughout the study.

The intervention period was 12 weeks. Study visits were conducted at baseline (week 0), week 4, week 8, and week 12. Recruitment was conducted from May to June 2023, and follow-up was performed from 1 July to 30 October 2023. No important changes to the trial methods or outcomes were made after trial commencement. Participant enrollment was performed by the operational coordinator, and allocation was conducted by the lead statistician. No patients were enrolled; all participants were community-dwelling Japanese adults residing in Hirosaki City, Aomori Prefecture, Japan. Patients and the public were not involved in the design, conduct, reporting, or dissemination plans of this research.

### 2.2. Intervention

Participants in the catechin + inulin group consumed one bottle per day of a test powdered beverage containing green tea catechins (400 mg/day) and water-soluble dietary fiber in the form of inulin (2.3 g/day), dissolved in 350 mL of water or hot water. Participants in the placebo group consumed a matched powdered beverage without catechins or inulin, dissolved in 350 mL of water or hot water. The powdered beverages were identical in appearance, taste, and pillows-style packaging, and were labeled with anonymized identification stickers. Participants were instructed to consume the powdered beverage, without a specified intake time, for 12 consecutive weeks, and to maintain their usual diet and physical activity during the study. The 12-week intervention period was selected because previous catechin intervention trials assessing visceral fat and metabolic outcomes have commonly used a similar duration. The catechin dose (400 mg/day) was chosen to fall within the range used in prior human studies, whereas the inulin dose (2.3 g/day) was selected as a practical, beverage-compatible amount intended for feasible long-term daily intake.

Adherence was monitored by counting returned pillow packs and by self-reported intake records. Participants were asked to avoid initiating new supplements or medications that could influence weight, lipid profile, or glucose metabolism during the trial.

### 2.3. Outcomes and Measurements

The primary outcome was the absolute change in VFA from baseline (week 0) to week 12. VFA was estimated by the abdominal bio-impedance method using EW-FA90 (Panasonic Corporation, Osaka, Japan), an approved medical device in Japan (No. 22500BZX00522000). Measurement values from this device have been reported to have a strong correlation with those obtained using CT, which is the gold standard for measuring visceral fat.

Secondary outcomes included body weight, BMI, waist circumference, systolic and diastolic blood pressure (SBP and DBP), fasting plasma glucose (Glu), HbA1c, fasting immunoreactive insulin (IRI), lipid profile (triglycerides [TG], HDL cholesterol [HDL-C], and LDL cholesterol [LDL-C]), liver enzymes (aspartate aminotransferase [AST], alanine aminotransferase [ALT], and gamma-glutamyl transferase [GGT]), liver fat assessed by CAP, and gut microbiota composition at the genus level.

Body weight and waist circumference were measured in light clothing without shoes. BMI was calculated as weight (kg) divided by height squared (m^2^). Blood pressure was measured in the seated position after at least 5 min of rest using an automated sphygmomanometer. Fasting blood samples were drawn in the morning after an overnight fast and analyzed at a certified laboratory using standard methods.

HOMA-IR was calculated as: HOMA-IR = [fasting glucose (mg/dL) × fasting insulin (µU/mL)]/405.

CAP, an index of liver steatosis, was measured using FibroScan^®^ (Echosens, Paris, France) with a transient elastography probe according to standard procedures. Examinations were considered unreliable and excluded if fewer than 10 valid measurements were obtained or if measurement quality criteria were not met.

### 2.4. Gut Microbiota Analysis

Stool samples were collected at baseline and week 12 in Metabolokeeper^®^ (TechnoSuruga Laboratory Co., Ltd., Shizuoka, Japan) [14]. After collection, samples were homogenized in the buffer and stored at 4 °C until DNA extraction.

Microbial community profiling was performed by amplicon sequencing of the V3–V4 region of the prokaryotic 16S rRNA gene. Briefly, DNA was extracted from the stool suspensions, and the V3–V4 region was amplified using universal primers that allow simultaneous detection of Bacteria and Archaea, as developed and validated by Takahashi et al. [15]. Paired-end sequencing was conducted on an Illumina MiSeq platform (Illumina, San Diego, CA, USA), following a workflow similar to that used in previous Japanese microbiome studies [16].

Raw demultiplexed paired-end 16S rRNA gene reads were processed in QIIME 2 (version 2024.2). Primer removal, quality filtering, dereplication, paired-end merging, and denoising (including chimera removal) were performed with DADA2 (dada2 denoise-paired). Resulting amplicon sequence variants (ASVs) were taxonomically classified using a Greengenes 13_8 (gg-13-8-99) Naive Bayes classifier. For downstream analyses, ASV counts were collapsed to genus level and converted to relative abundance per sample; relative abundance was calculated as the proportion of reads assigned to each taxon out of the total reads in that sample.

### 2.5. Sample Size Calculation

The required sample size was estimated based on the expected between-group difference in the 12-week change in VFA. A prior pooled analysis of catechin intervention trials in Japanese adults [4] suggested that catechin intake could reduce VFA by approximately 9.5 cm^2^ relative to controls, with a standard deviation of approximately 16.8 cm^2^. Assuming a between-group difference of 9.51 cm^2^, a common SD of 16.81 cm^2^, a two-sided α of 0.05, and 80% power, we calculated that 51 participants per group (102 total) would be required. Allowing for an anticipated dropout rate of 10%, we set a planned enrollment target of 57 participants per group (114 total). However, owing to recruitment feasibility and time constraints, enrollment was stopped at 96 randomized participants. Under the original assumptions, this sample size corresponds to an estimated power of approximately 78%.

### 2.6. Statistical Analysis

The primary analysis population was the full analysis set (FAS), defined as all randomized participants who consumed at least one dose of the study beverage and had VFA measured at both baseline and week 12.

Continuous variables are presented as mean ± standard deviation (SD) or median (interquartile range [IQR]), as appropriate, and categorical variables are shown as numbers and percentages. For efficacy outcomes, the primary analysis compared the absolute change from baseline to week 12 between the two groups. We additionally performed exploratory sex-stratified analyses for key outcomes. For the primary endpoint VFA and other anthropometric parameters (body weight, BMI, waist circumference, systolic blood pressure, and diastolic blood pressure), between-group differences in 12-week changes were evaluated using Student’s *t*-test, and these results are illustrated as box-and-whisker plots. For blood-based biomarkers (fasting glucose, HbA1c, triglycerides, HDL-C, LDL-C, AST, ALT, GGT, fasting immunoreactive insulin, and HOMA-IR), summary statistics indicated that many change values were skewed; therefore, as a more conservative and exploratory approach, we used the Mann–Whitney U test to compare 12-week changes between groups.

Gut microbiota analyses focused on genus-level relative abundances for 104 genera. For each genus, the between-group difference in change from baseline to week 12 was assessed using the Mann–Whitney U test. Raw *p*-values were adjusted for multiple comparisons using the Benjamini–Hochberg method to control the false discovery rate (FDR). We considered FDR < 0.05 as statistically significant after correction. Exploratory correlation analyses were conducted to examine the relationships between changes in selected genera of interest (e.g., *Coprococcus*, *Bifidobacterium*) and changes in HOMA-IR, using Pearson correlation coefficients.

All statistical analyses were performed using R software (version 4.1.1; R Foundation for Statistical Computing, Vienna, Austria). Two-sided *p*-values < 0.05 were considered statistically significant.

### 2.7. Ethics

The study was conducted in accordance with the Declaration of Helsinki and was approved by the Certified Review Board of Hirosaki University Hospital (approval no. 2022-A-003; approval date: 25 April 2023) and by the Ethics Committee of the Hirosaki University Graduate School of Medicine (approval no. 2024-155; approval date: 26 February 2025). All participants provided written informed consent prior to enrollment. The participant information sheet and written informed consent form were provided to all participants, and written informed consent was obtained (signed) before any study procedures.

## 3. Results

### 3.1. Participant Flow and Baseline Characteristics

**Figure 1:**
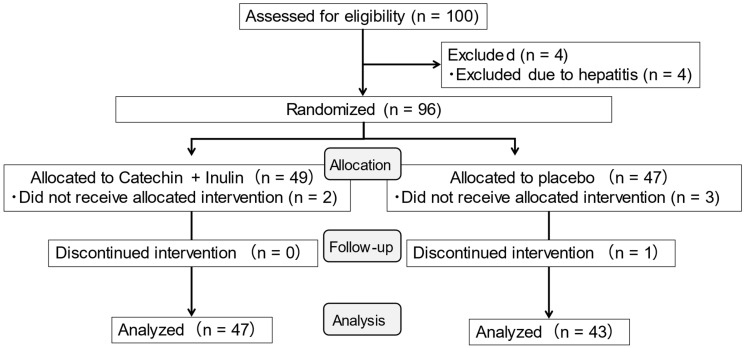
Flow diagram of participant enrollment, randomization, follow-up, and analysis.

Of the 96 participants randomized (catechin + inulin group, n = 49; placebo group, n = 47), two participants in the catechin + inulin group and three in the placebo group discontinued the study for personal reasons during the intervention period. VFA measurements at both baseline and week 12 were available for 47 participants in the catechin + inulin group and 44 participants in the placebo group, comprising the FAS for the primary outcome (Figure 1).

Baseline characteristics of the FAS are summarized in Table 1. The two groups were generally well balanced at baseline with respect to age, sex distribution, VFA, body weight, BMI, waist circumference, SBP, DBP, fasting glucose, HbA1c, lipid profile (TG, HDL-C, and LDL-C), and liver enzymes (AST, ALT, and GGT). There were no significant between-group differences in these baseline variables. Adherence to the study beverages was high in both groups, and the number of participants with intake rates below 80% was small.

Regarding safety, the overall incidence of adverse events was similar between groups, and most events were mild and resolved spontaneously. No serious adverse events with a plausible relationship to the study beverages were observed, and no participant discontinued the study because of safety concerns. AST and ALT showed small increases in the catechin + inulin group compared with the placebo group; although these differences were statistically significant, all values remained within the normal range, and no clinically meaningful hepatic or renal impairment was detected.

### 3.2. Changes in Visceral Fat Area and Anthropometric Parameters

**Figure 2:**
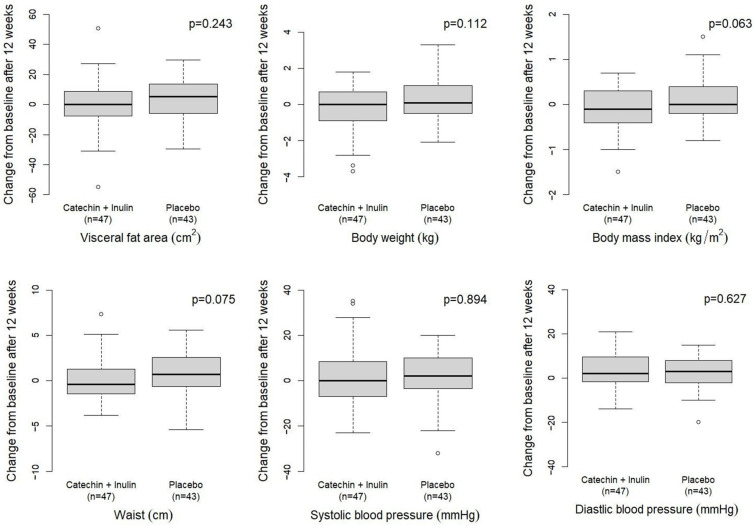
Comparison of changes in anthropometric variables between treatment groups. Box-and-whisker plots of 12-week changes in visceral fat area (VFA), body weight, BMI, and waist circumference by group. Between-group differences in changes were evaluated using Student’s *t*-test; *p* values are shown in each panel.

Changes in VFA and other anthropometric measures from baseline to week 12 are presented in Table 2. The primary outcome, VFA, increased slightly in both groups over 12 weeks, with a mean change of +0.91 cm^2^ in the catechin + inulin group and +4.61 cm^2^ in the placebo group. The between-group difference in VFA change was not statistically significant (*p* = 0.243), indicating that the intervention did not produce a clear reduction in VFA compared with placebo under the present conditions (Figure 2).

Body weight, BMI, and waist circumference showed small decreases in the catechin + inulin group and small increases in the placebo group, but the between-group differences in changes were not statistically significant. Similarly, SBP and DBP did not differ significantly between groups in terms of 12-week changes (Figure 2). In sex-stratified analyses, women showed a numerically greater decrease in VFA, whereas men showed numerically greater decreases in body weight and BMI; however, none of the between-group differences reached statistical significance (Supplementary Table S1).

### 3.3. Changes in Metabolic and Liver-Related Parameters

Changes in metabolic parameters and liver-related indices are shown in Table 2. LDL-C tended to decrease slightly in the catechin + inulin group and increase in the placebo group, but the between-group difference did not reach statistical significance. TG and HDL-C also showed no significant between-group differences in change.

AST and ALT increased modestly in the catechin + inulin group, whereas they remained relatively stable in the placebo group, resulting in statistically significant between-group differences. However, these changes were small in magnitude and remained within the normal reference ranges, suggesting limited clinical relevance. GGT and other liver and renal function markers did not show notable or clinically concerning between-group differences.

CAP, an index of liver fat, changed by −2.0 ± 58.5 dB/m (n = 44) in the catechin + inulin group and by −6.8 ± 64.1 dB/m (n = 42) in the placebo group over 12 weeks, with no significant between-group difference (*p* = 0.72).

### 3.4. Changes in Insulin Resistance (HOMA-IR)

HOMA-IR was evaluated as a key marker of insulin resistance. Baseline HOMA-IR was comparable between groups. From baseline to week 12, HOMA-IR decreased in the catechin + inulin group and increased in the placebo group (mean change: −0.32 ± 1.71 vs. +0.18 ± 0.76), with a statistically significant between-group difference (*p* = 0.020).

Fasting IRI also tended to decrease in the catechin + inulin group and increase in the placebo group (mean change: −1.05 ± 5.55 vs. +0.73 ± 2.59), (*p* = 0.009). In contrast, fasting glucose and HbA1c exhibited minimal changes in both groups, with no significant between-group differences.

### 3.5. Changes in Gut Microbiota and Associations with HOMA-IR

Genus-level gut microbiota profiles were available for 104 genera. Between-group comparisons of changes in relative abundance from baseline to week 12 were performed, and the 10 genera with the smallest raw *p*-values are summarized in Table 3. The genus with the smallest *p*-value for between-group difference in change was *Coprococcus*, followed by *Parabacteroides*, *Oxalobacter*, *Holdemania*, *Fusobacterium*, *Bifidobacterium*, and others. However, after Benjamini–Hochberg FDR correction for multiple comparisons, no genus achieved FDR < 0.05, and thus no taxa showed statistically significant between-group differences in change when accounting for multiple testing.

**Figure 3:**
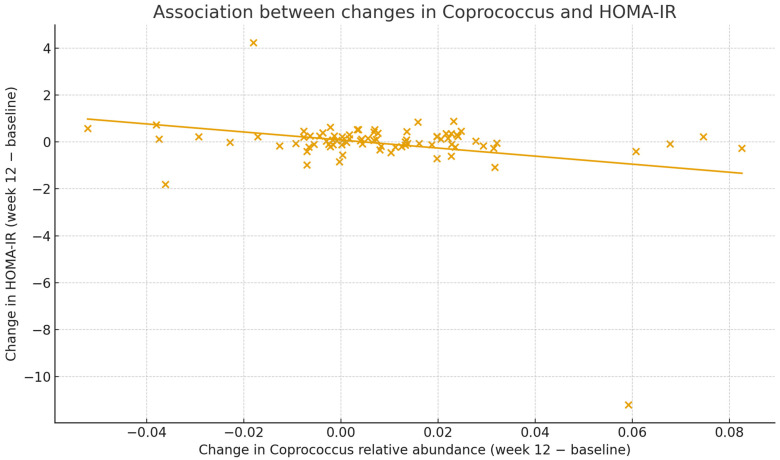
Association between changes in *Coprococcus* relative abundance and insulin resistance. Scatter plot showing the relationship between the 12-week change (Week 12 minus Week 0) in genus-level relative abundance of *Coprococcus* and the corresponding 12-week change in HOMA-IR. The solid line represents the fitted linear regression line. An inverse correlation was observed (Pearson’s r = −0.28, *p* = 0.010). HOMA-IR was calculated as fasting glucose (mg/dL) × fasting insulin (µU/mL)/405. See Table S2 for Pearson correlation results and Figure S1 for the volcano plot of genus-level between-group differences.

To further explore the relationship between gut microbiota changes and insulin resistance, we conducted correlation analyses across all participants with available microbiota and HOMA-IR data at both time points (n = 83). We focused on *Coprococcus* and *Bifidobacterium*, which showed relatively small *p*-values in between-group comparisons and have been implicated in metabolic health in previous studies. The change in *Coprococcus* relative abundance from baseline to week 12 was inversely correlated with the change in HOMA-IR (r = −0.28, *p* = 0.010), indicating that participants with greater increases in *Coprococcus* tended to exhibit greater improvements in HOMA-IR (Figure 3). In contrast, the change in *Bifidobacterium* relative abundance was not clearly associated with the change in HOMA-IR (r = 0, *p* = 0.95).

Taken together, these findings suggest that, although no genus demonstrated a statistically significant between-group change after multiple testing correction, the butyrate-producing genus *Coprococcus* may be relevant to the observed improvement in insulin resistance in this trial. To provide an overview of the distribution of genus-level changes, a volcano plot of between-group differences is shown in Figure S1. Pearson correlation coefficients between changes in HOMA-IR and changes in selected genera are summarized in Table S2.

Importantly, no genus remained significant after FDR correction, indicating that there were no robust between-group differences in genus-level changes. Therefore, these microbiota results should be interpreted as exploratory signals rather than as evidence of a causal mechanism.

## 4. Discussion

In this double-blind randomized controlled trial involving Japanese adults with visceral obesity, daily intake of a beverage containing green tea catechins (400 mg/day) and inulin (2.3 g/day) for 12 weeks did not significantly reduce VFA compared with the placebo, and thus the primary endpoint was not achieved. However, the intervention was associated with a significant improvement in insulin resistance as assessed by HOMA-IR, with HOMA-IR decreasing in the catechin + inulin group and slightly increasing in the placebo group. Exploratory analyses of the gut microbiota revealed that changes in the relative abundance of the butyrate-producing genus *Coprococcus* were inversely correlated with changes in HOMA-IR, suggesting a possible role of specific gut bacteria in mediating the metabolic effects of the intervention.

HOMA-IR is a widely used surrogate marker of insulin resistance, calculated from fasting glucose and insulin, and has been associated with incident cardiovascular events in adults without diabetes [17]. Several RCTs and meta-analyses have examined the effects of green tea or catechins on glycemic control and insulin resistance. In Japanese individuals with impaired glucose tolerance, high-dose green tea polyphenol intake has been reported to improve HOMA-IR and inflammatory markers [6]. A meta-analysis of RCTs in patients with type 2 diabetes suggested that green tea consumption may modestly reduce fasting glucose, HbA1c, and HOMA-IR [7]. Our study extends these findings by demonstrating that, even in non-diabetic adults with visceral obesity, a catechin-containing beverage in combination with inulin can improve insulin resistance, despite the lack of a clear effect on visceral fat within the 12-week intervention period.

The absence of a significant effect on VFA in the present study contrasts with previous trials in which catechin-rich green tea beverages reduced VFA and abdominal obesity in Japanese populations [3,4,5]. Several factors may explain this discrepancy. First, the dose of inulin used in our trial (2.3 g/day) was relatively low compared with many inulin or ITF trials, which have often used ≥10 g/day and shown more robust effects on body weight, adiposity, and glycemic control [8,9]. This specific dose of 2.3 g/day was selected to ensure safety and prevent gastrointestinal side effects associated with long-term daily consumption, while anticipating a synergistic effect with the concurrent intake of green tea catechins. However, considering the lack of a significant reduction in VFA, it is highly possible that a dose–response relationship exists for inulin. Higher doses may be required to observe more pronounced metabolic benefits, even when combined with catechin. Second, while the catechin dose in this study (400 mg/day) falls within the lower range of doses used in earlier trials, several positive studies employed higher doses of catechins (e.g., 500–600 mg/day or more) and sometimes combined catechin intake with additional lifestyle interventions such as caloric restriction or exercise [3,4,5]. Thus, both the relatively modest doses of inulin and catechins and the free-living conditions may have attenuated the impact on VFA in the current study.

The Taiwanese RCT by Yang et al. demonstrated that a 6-week intake of a drink combining catechin-rich green tea and inulin resulted in significant reductions in body weight and body fat, with some persistence of the effect after a 2-week washout [12]. That study focused on body composition and did not comprehensively evaluate VFA, insulin resistance, or gut microbiota. Our trial adds to the field by extending the intervention duration to 12 weeks and assessing VFA, HOMA-IR, CAP, and gut microbiota simultaneously. Nevertheless, the relatively low dose of inulin and the limited sample size may have reduced the power to detect small to moderate effects on visceral adiposity.

In the exploratory gut microbiota analyses, no genus exhibited a statistically significant between-group difference in change after FDR correction, which is unsurprising given the modest sample size and the large number of taxa tested. However, *Coprococcus* and *Bifidobacterium* showed trends toward increased relative abundance in the catechin + inulin group before correction. Moreover, when all participants were analyzed together, increases in *Coprococcus* were associated with reductions in HOMA-IR. *Coprococcus* belongs to the family Lachnospiraceae and is recognized as a butyrate-producing genus. Butyrate has multiple beneficial effects, including serving as an energy source for colonocytes and activating GPR41/43, which promotes GLP-1 secretion, enhances hepatic fatty acid oxidation, and improves insulin sensitivity [10]. Previous studies have reported that lower abundances of butyrate-producing bacteria and reduced SCFA levels are associated with obesity, insulin resistance, and metabolic syndrome [10,11], whereas dietary fiber interventions that increase SCFA production can ameliorate these conditions [8,9,10].

Our findings are consistent with the hypothesis that modulation of SCFA-producing bacteria, including *Coprococcus*, may contribute to improved insulin sensitivity. Recent integrative analyses have further suggested that the carbohydrate-metabolizing capacity of gut microbiota is linked to insulin resistance and non-alcoholic fatty liver disease in humans [18]. In this context, the observed association between *Coprococcus* and HOMA-IR improvement provides a mechanistic clue that the combination of catechins and inulin might act, at least in part, through changes in specific gut microbiota taxa and their metabolic outputs. Recent studies have also highlighted that tea polyphenols and polysaccharides can modulate gut microbiota, promote SCFA production, and improve intestinal barrier function to alleviate obesity and metabolic disorders [19,20]. Furthermore, natural plant-derived formulas have been shown to regulate fecal microbiota and their metabolites [21]. These findings further support the biological plausibility of our observation. Because this study was not designed to test mediation and did not control dietary intake, the observed associations cannot be used to infer that microbiota changes caused the improvement in HOMA-IR. Nonetheless, it is important to emphasize that these microbiota findings are exploratory, and the correlation between *Coprococcus* and HOMA-IR does not establish causality.

### Limitations

This study has several limitations. First, the sample size was smaller than originally planned, leading to limited statistical power to detect modest differences in VFA and other secondary endpoints. Based on the effect size assumptions from previous pooled analyses [4], our final sample size provided an estimated power of approximately 78% for the primary endpoint. Because this is slightly below the conventional 80% threshold, we cannot rule out the possibility of a Type II error; if the actual effect size of the intervention was smaller than initially assumed, true differences in VFA might have been undetected. Second, the dose of inulin was substantially lower than in many prior ITF trials [8,9], and the catechin dose was at the lower end of the effective range reported for visceral fat reduction [3,4,5]. Third, the intervention period was relatively short (12 weeks); longer-term interventions might be necessary to observe more pronounced changes in visceral fat, liver fat, and gut microbiota composition. Fourth, the gut microbiota analyses were exploratory and involved multiple comparisons, resulting in a lack of significant taxa after FDR correction. Dietary intake was not strictly controlled and was assessed under free-living conditions; thus, residual dietary variation could have influenced the gut microbiota. In addition, the microbiota analyses were exploratory and may have been underpowered to detect subtle but consistent between-group differences. Accordingly, the findings, particularly those for secondary outcomes and exploratory microbiota analyses, should be interpreted with appropriate caution. Despite these limitations, the present trial has several strengths. It employed a double-blind randomized design, targeted adults with objectively measured visceral obesity, and simultaneously evaluated VFA, insulin resistance, liver fat, and gut microbiota. The observed improvement in HOMA-IR, together with the exploratory association between *Coprococcus* changes and HOMA-IR, suggests that even a relatively low dose of inulin combined with green tea catechins may influence insulin resistance in adults with visceral obesity, possibly via alterations in SCFA-producing gut bacteria.

## 5. Conclusions

In this double-blind randomized controlled trial involving Japanese adults with visceral obesity, a 12-week intake of a beverage containing green tea catechins (400 mg/day) and inulin (2.3 g/day) did not significantly reduce the visceral fat area compared with the placebo. Insulin resistance assessed by HOMA-IR improved; however, this was a secondary endpoint and should be interpreted cautiously. Exploratory analyses suggested an inverse association between changes in Coprococcus and changes in HOMA-IR; however, no taxa remained significant after FDR correction, and these microbiota findings are hypothesis-generating. Overall, these results suggest that this practical dietary intervention may have clinical relevance for insulin resistance and warrant larger, adequately powered trials to confirm the metabolic effects and clarify microbiota-related mechanisms.

## Figures and Tables

**Table 1:** Baseline characteristics of participants in the full analysis set (FAS).

Variable	Catechin + Inulin (n = 47) Median (IQR)	Placebo (n = 44) Median (IQR)	*p* Value
Sex: female ^1^	45% (21)	43% (19)	1 ^2^
Age (years)	57.0 (44.5, 64.5)	56.5 (45.8, 66.0)	0.947
Visceral fat area, VFA (cm^2^)	108.7 (93.7, 138.7)	112.8 (94.8, 140.1)	0.780
Body weight (kg)	70.0 (65.4, 77.1)	70.1 (65.0, 76.1)	0.495
BMI (kg/m^2^)	26.9 (25.2, 28.5)	26.1 (24.4, 27.9)	0.379
Waist circumference (cm)	91.4 (88.4, 95.7)	91.6 (87.9, 97.8)	0.783
Systolic blood pressure (mmHg)	133.0 (122.0, 145.0)	123.0 (116.8, 143.5)	0.157
Diastolic blood pressure (mmHg)	82.0 (73.5, 87.5)	79.5 (74.8, 87.3)	0.667
Fasting glucose (mg/dL) *	91.0 (85.3, 97.0)	92.0 (87.8, 96.3)	0.588
HbA1c (%)	5.5 (5.3, 5.7)	5.4 (5.3, 5.7)	0.483
Triglycerides, TG (mg/dL) *	123.5 (90.0, 198.0)	133.0 (87.3, 159.8)	0.503
HDL cholesterol (mg/dL) *	55.0 (47.0, 61.0)	55.0 (48.8, 71.5)	0.329
LDL cholesterol (mg/dL) *	125.5 (106.5, 145.8)	130.5 (110.0, 153.0)	0.561
AST (U/L)	20.0 (18.0, 25.5)	22.0 (19.0, 28.0)	0.267
ALT (U/L)	23.0 (16.5, 31.5)	22.5 (17.8, 28.3)	0.922
γ-GTP (U/L)	32.0 (22.0, 49.0)	31.5 (21.0, 50.0)	0.774
Insulin, IRI (µU/mL) *	5.6 (4.4, 7.5)	5.4 (3.9, 7.2)	0.461
HOMA-IR *	1.3 (1.0, 1.8)	1.3 (0.9, 1.7)	0.734

^1^, Numbers after percents are frequencies. ^2^, Fisher exact test; otherwise, Mann–Whitney U test. * One subject in the catechin + inulin group consumed breakfast at week 0, and therefore data were omitted (n = 46). IQR, Interquartile range.

**Table 2:** Changes in metabolic parameters from baseline (Week 0) to Week 12 in the full analysis set (FAS).

	Catechin + Inulin		Placebo		
Variable	Change (Week 12–Week 0) Mean ± SD		Change (Week 12–Week 0) Mean ± SD		Mann–Whitney U-Test; *p* Value
Fasting glucose (mg/dL)	n = 46 *	−0.39 ± 6.20	n = 42	0.31 ± 6.05	1.000
HbA1c (%)	n = 47	0.09 ± 0.18	n = 42	0.15 ± 0.24	0.433
Triglycerides, TG (mg/dL)	n = 46 *	−20.89 ± 158.68	n = 42	−8.48 ± 60.38	0.957
HDL cholesterol (mg/dL)	n = 46 *	0.46 ± 6.56	n = 42	−1.36 ± 6.79	0.252
LDL cholesterol (mg/dL)	n = 46 *	−3.78 ± 20.66	n = 42	3.98 ± 22.65	0.167
AST (U/L)	n = 47	4.49 ± 12.90	n = 42	−0.88 ± 8.74	0.004
ALT (U/L)	n = 47	5.55 ± 11.47	n = 42	−0.07 ± 12.13	0.053
GGT (U/L)	n = 47	2.81 ± 15.33	n = 42	2.93 ± 15.23	0.805
Insulin, IRI (µU/mL)	n = 46 *	−1.05 ± 5.55	n = 42	0.73 ± 2.59	0.009
HOMA-IR	n = 46 *	−0.32 ± 1.71	n = 42	0.18 ± 0.76	0.020

One subject in the placebo group was not visited at Week 12. * One subject in the catechin + inulin group consumed breakfast at week 0, and therefore data were omitted.

**Table 3:** Top 10 genera ranked by unadjusted *p*-values for between-group differences in 12-week changes in relative abundance (Week 12–Week 0). Unadjusted *p*-values were calculated using the Mann–Whitney U test. Multiple testing was addressed using the Benjamini–Hochberg false discovery rate (FDR), reported as q values.

Genus	*p*-Value	q-Value
*Coprococcus*	0.0016	0.1699
*Parabacteroides*	0.0232	1
*Oxalobacter*	0.0232	0.8057
*Holdemania*	0.0298	0.7761
*Fusobacterium*	0.0355	0.7374
*Bifidobacterium*	0.0422	0.7318
*Anaerococcus*	0.0481	0.7149
*Prevotella*	0.0532	0.6912
*Lactobacillus*	0.0544	0.6291
*Akkermansia*	0.0547	0.5692

Top genera showing the smallest between-group *p*-values for the 12-week change in relative abundance. Unadjusted *p*-values were calculated using the Mann–Whitney U test, and multiple testing was addressed using the Benjamini–Hochberg false discovery rate (FDR). Although *Coprococcus* showed the lowest unadjusted *p*-value (*p* = 0.0016), no genus reached significance after FDR correction (all q-values > 0.05).

## Data Availability

The data presented in this study are not publicly available due to privacy and ethical restrictions but are available from the corresponding author on reasonable request.

## Supplementary Materials

The following supporting information can be downloaded at: https://www.mdpi.com/article/10.3390/nu18050851/s1.

## Abbreviations

The following abbreviations are used in this manuscript:

ALT	alanine aminotransferase
AST	aspartate aminotransferase
BMI	body mass index
CAP	controlled attenuation parameter
DBP	diastolic blood pressure
FAS	full analysis set
FDR	false discovery rate
GGT	gamma-glutamyl transferase
Glu	fasting plasma glucose
HbA1c	glycated hemoglobin
HDL-C	high-density lipoprotein cholesterol
HOMA-IR	homeostasis model assessment of insulin resistance
IQR	interquartile range
IRI	immunoreactive insulin (fasting insulin)
LDL-C	low-density lipoprotein cholesterol
RCT	randomized controlled trial
r	Pearson’s correlation coefficient
SBP	systolic blood pressure
SCFAs	short-chain fatty acids
SD	standard deviation
TG	triglycerides
VFA	visceral fat area
wk	week(s)
