# Supplementary figures and images for: Green Tea Catechin Plus Inulin Improves Insulin Resistance Without Reducing Visceral Fat and Shows Exploratory Gut Microbiota Signals in Adults with Visceral Obesity: A Double-Blind Randomized Controlled Trial
Source: Nutrients. 2026 Mar 6;18(5):851. doi: 10.3390/nu18050851 (PMC12986909; doi:10.3390/nu18050851)

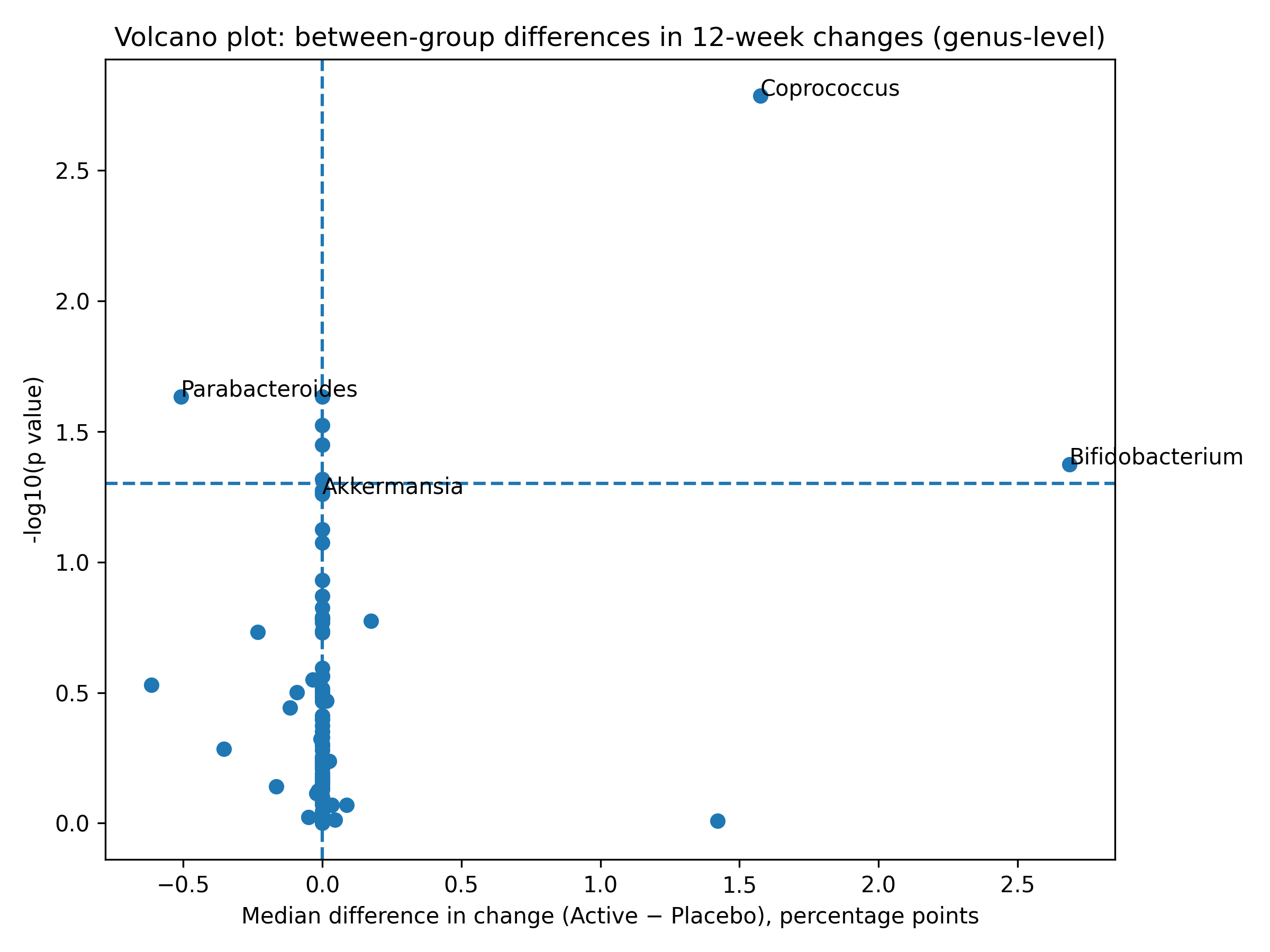

Supplement: Supplementary file 1 [file nutrients-18-00851-s001.zip › nutrients-4149467-Figure S1.png]
